# Exploring Acceptance of Digital Health Technologies for Managing Non-Communicable Diseases Among Older Adults: A Systematic Scoping Review

**DOI:** 10.1007/s10916-025-02166-3

**Published:** 2025-03-11

**Authors:** Sadia Azmin Anisha, Arkendu Sen, Badariah Ahmad, Chris Bain

**Affiliations:** 1 Jeffrey Cheah School of Medicine & Health Sciences, Monash University Malaysia, Bandar Sunway, Subang Jaya, Selangor Malaysia; 2 Faculty of Information Technology, Monash University, Clayton, Melbourne, VIC Australia

**Keywords:** Technology acceptance, Digital health, Artificial intelligence, Conversational agents, Older adults, Non-communicable disease management

## Abstract

This review explores the acceptance of digital health (DH) technologies for managing non-communicable diseases (NCDs) among older adults (≥ 50 years), with an extended focus on artificial intelligence (AI)-powered conversational agents (CAs) as an emerging notable subset of DH. A systematic literature search was conducted in June 2024 using PubMed, Web of Science, Scopus, and ACM Digital Library. Eligible studies were empirical and published in English between January 2010 and May 2024. Covidence software facilitated screening and data extraction, adhering to PRISMA-ScR guidelines. The screening process finally yielded 20 studies. Extracted data from these selected studies included interventions, participant demographics, technology types, sample sizes, study designs and locations, technology acceptance measures, key outcomes, and methodological limitations. A narrative synthesis approach was used for analysis, revealing four key findings: (1) overall positive attitudes of older adults towards DH acceptance; (2) the Technology Acceptance Model (TAM) and the Unified Theory of Acceptance and Use of Technology (UTAUT) are the most frequently used standard frameworks for evaluating technology acceptance; (3) the key facilitators of technology acceptance include *perceived usefulness*, *ease of use*, *social influence*, and *digital* or *e-health literacy*, while barriers involve *technical challenges*, *usability issues*, and *privacy concerns*; (4) the acceptance of AI-based CAs for NCD management among older adults remains inadequately evaluated, possibly due to limited adaptation of established frameworks to specific healthcare contexts and technology innovations. This review significantly contributes to the DH field by providing a comprehensive analysis of technology acceptance for NCD management among older adults, extending beyond feasibility and usability. The findings offer stakeholders valuable insights into how to better integrate these technologies to improve health outcomes and quality of life for older adults. Protocol Registration: PROSPERO (Registration ID: CRD42024540035).

**Supplementary Information:**

The online version contains supplementary material available at 10.1007/s10916-025-02166-3.

## Introduction

### Prevalence of Non-Communicable Diseases Among Older Adults

Non-communicable diseases (NCDs) such as cardiovascular diseases (CVDs), metabolic syndrome (MetS), diabetes, hypertension, chronic respiratory and kidney diseases, and cancers are chronic diseases that are not transmitted across individuals through infections [1–3]. They are characterized by long durations and generally slow progressions, posing significant health challenges, especially among older adults [1].

Age is a critical factor in the risk of developing NCDs due to a combination of biological, behavioral, and environmental factors [4, 5]. The increasing prevalence of NCDs in the growing aging population is a pressing global health concern, particularly in low- and middle-income countries (LMICs), where the burden is disproportionately high [6]. By 2050, the global population of individuals aged ≥ 65 years is projected to double [7], which is expected to substantially increase the burden of NCDs responsible for premature deaths in older adults aged < 70 years [1]. Alarmingly, 86% of these premature mortalities occur in LMICs [1].

The high prevalence of NCDs in older populations can be largely attributed to lifestyle choices, such as physical inactivity, poor diet, tobacco use, and excessive alcohol consumption [3]. These modifiable behavioral factors, and unmodifiable physiological changes associated with aging, exacerbate the risk of developing NCDs [1, 3]. Moreover, environmental and social determinants such as urbanization, socioeconomic status, and access to healthcare further influence health outcomes for older adults globally [8]. Therefore, addressing this rising health burden requires a coordinated approach, targeting effective NCD management strategies for the aging population [2, 6].

### The Role of Technology in NCD Management

In response to the challenges posed by NCDs, technology is increasingly recognized as a vital tool for enhancing chronic disease management, particularly for older adults, who may face barriers to accessing traditional healthcare services. Digital health (DH) interventions encompass a wide range of digital tools (e.g., e-health apps, telemonitoring systems, wearable devices, etc.), which have shown promise in enhancing the management of NCDs by improving accessibility, patient engagement, and health outcomes [9].

Telehealth services have expanded substantially, especially during the COVID-19 pandemic, driven by rapid technological advancements and widespread adoption of mobile devices, allowing patients to access healthcare remotely [10]. This approach addresses the mobility challenges older adults encounter, while also reducing hospital visits and conserving resources for critical health conditions and emergencies. As older adults experience social isolation due to younger family members relocating for education, work, or other reasons, the need for remote care is growing [11, 12], making telehealth a viable solution [2]. DH tools facilitate continuous monitoring of health indicators through wearable devices and mobile applications, enabling timely interventions and personalized care plans tailored to individual health needs [2, 10].

Building on the foundation established by telehealth, advanced artificial intelligence (AI)-assisted technologies in the form of conversational agents (CAs), such as chatbots *(e.g., ChatGPT)*, voicebots *(e.g., Amazon Alexa)*, and digital human-like avatars *(e.g., Replika)* [2], represent an emerging subset of DH innovations that can further enhance telehealth experience for older adults, especially those with limited technological experience or disabilities [2, 13, 14]. These agents, designed to mimic human-like interaction, can provide personalized health advice in simplified language, thereby improving usability and patient engagement [2, 15]. Prior research has demonstrated the potential effectiveness of AI-based CAs in managing chronic conditions, such as atrial fibrillation (AF) and type 2 diabetes mellitus (T2DM), with significant improvements in quality of life (*P* < 0.05) among older adults [2, 16–18]. Therefore, integrating AI CAs as virtual health caregivers into healthcare delivery can improve remote care accessibility and strengthen health management strategies for older adults with NCDs [2].

### State-of-the-Art-Summary

The recent developments in AI-assisted telehealth, particularly through AI-based CAs, offer promising avenues for enhancing chronic disease management through personalized and timely intervention in human-like manner [2]. Nevertheless, the acceptance of such modern technologies for managing NCDs among older adults remains underexplored. While existing systematic and scoping reviews have addressed DH adoption barriers and facilitators for older adults with chronic diseases or multimorbidity [19–21], none, to the best of our knowledge, has explicitly focused on the acceptance of DH, especially AI-based CAs, for NCD management in this demographic. Our recent scoping review on AI-powered CAs for remote management of NCDs [2] predominantly identified conventional feasibility and usability studies that primarily emphasize design factors influencing user-friendliness and user experience [22, 23], underscoring substantial inadequacy in acceptability evaluations of AI CAs, which assess users’ intention to adopt or utilize a digital tool for its intended purpose [22, 24]. This gap inspired the present review, focusing on technology acceptance aspects of DH in the NCD management context for older adults. The findings of this review present both challenges and opportunities for advancing DH research in geriatric NCD care.

### Objective

This systematic scoping review aims to explore the acceptance of different DH technologies, with extended emphasis on AI-powered CAs, regarded as a prominent subset of DH. The review seeks to understand the trends and the growing adoption of these emerging tools for self-management of NCDs in older adults (≥ 50 years), providing a comprehensive overview of perceived acceptability, key influential factors, methodological frameworks, acceptance evaluation measures employed to determine users’ intention to use a technology (e.g., perceived usefulness, ease of use, performance expectancy, effort expectancy, etc.), followed by implications for future research and practice in this domain. By examining the current literature on this topic, we intended to identify key areas for further investigations and provide insights to facilitate technology acceptance in the aging population for improved health outcomes.

### Research Questions

#### Overall DH Acceptability:

i) What is the current perceived acceptability of various DH technologies, especially AI-based CAs, among older adults for managing different types of NCDs?
ii) Does technology acceptance vary across different NCDs?

#### Theoretical Frameworks and Corresponding Influential Factors:

iii) Which models or frameworks are commonly used to assess technology acceptance in this context?
iv) What factors based on the frameworks used, may influence acceptance of such technologies for managing NCDs among older adults?

## Methods

The review utilized a scoping review approach, following PRISMA Extension for Scoping Reviews (PRISMA-ScR) guidelines [25], to encompass a comprehensive scope and examine diverse evidence from various study designs and methodologies in the literature [26]. Our review protocol was prospectively registered in PROSPERO *(Registration ID: CRD42024540035)* on May 13, 2024 [27].

### Search Strategy

A comprehensive search strategy guided by the SPIDER framework (Sample, Phenomenon of Interest, Design, Evaluation, Research type) [28] was established without imposing restrictions on research types to ensure the broad inclusion of studies exploring the acceptance of DH, including AI-based CAs among older adults for managing NCDs. Initially, research questions were formulated, followed by a systematic literature search conducted on June 1, 2024, using four major electronic databases: PubMed, Web of Science, Scopus, and the ACM Digital Library, employing various combinations of search terms associated with older adults, technology acceptance, digital health, AI, CAs and NCD management. These databases were selected for their extensive coverage of peer-reviewed literature on DH and AI technologies, which corresponds to our scoping review’s interdisciplinary scope spanning health sciences, technology, and social sciences. Scopus and Web of Science comprise diverse disciplines that support such interdisciplinary research focus. PubMed emphasizes medical and healthcare-related fields, encompassing research on DH interventions and NCD management. The ACM Digital Library offers a broad coverage of technology and human–computer interaction (HCI), including conference proceedings on innovative technologies, essential for capturing the latest advancements in DH and AI-based CAs. The comprehensive search strategy implemented using these databases was deemed sufficient for a thorough exploration of peer-reviewed literature, which aided in minimizing publication bias. Consequently, an additional gray literature search was not considered necessary.

The search query utilized a combination of keywords, such as “older adults”, “non-communicable diseases”, “self-manage”, “digital health”, artificial intelligence, and “technology acceptance”, including their associated synonyms and abbreviations (as shown in **Textbox 1**). The Boolean operators “*” and “OR” were applied to capture various word combinations, while the operator “AND” was used to refine the search to studies specifically addressing technology acceptance in the context of managing NCDs among older adults.

**Textbox 1** Search Query (PubMed)

**Table:** 

(“older adult*” OR “older patient*” OR “elderly” OR “geriatric*” OR “aging” OR “ageing” OR “senior*”)
AND (“health” OR “*care” OR “chronic disease*" OR “non-communicable disease*” OR “noncommunicable disease*”) AND (“self-manage*” OR “self-monitor*” OR “self-care” OR “self-treat*” OR “manage*” OR “monitor*” OR “control*” OR “prevent*” OR “treat*” OR “remote care” OR “intervention”) AND (“artificial intelligence” OR “intelligent” OR “AI” OR “AI-based” OR “AI-powered” OR “AI-assisted” OR “machine learning” OR “deep learning” OR “chatbot*” OR “conversational agent*” OR “virtual agent*” OR “virtual coach*” OR “virtual assistant*” OR “digital health” OR "internet*" OR "web" OR "mobile*" OR "smartphone*" OR "ubiquitous" OR “e-health” OR “e-Health” OR “mHealth” OR “mhealth” OR “m-health” OR “m-Health” OR "mobile health" OR “telehealth” OR “telemedicine”) AND (“technology” AND “acceptance”) OR (“technology” AND “adoption”) OR (“acceptance model” OR “technology acceptance”) OR (“TAM” OR “TAM2” OR “STAM” OR “UTAUT” OR “UTAUT2”)

### Eligibility Criteria

Our eligibility criteria (Table 1) targeted empirical studies on the acceptance of DH technologies and AI-based CAs for managing NCDs among older adults, including full-text scholarly articles in English published between January 2010 and May 2024. Exclusions included studies focusing on physically embodied robots, questionnaire development, usability evaluations without acceptance outcomes, social media or telephone-based interventions, and telehealth involving only human professionals without e-health apps were excluded. Other exclusions involved review articles, protocols, commentaries, studies on communicable diseases, younger populations, or non-NCD topics such as surgery, maternity, accidents, and loneliness.

**Table 1:** The inclusion and exclusion criteria

Inclusion Criteria	Exclusion Criteria
Studies including or focusing on older adults (≥ 50 years)	Studies limited to younger populations (< 50 years)
At least one study objective was to evaluate technology acceptance of a DH^a^ technology or an AI^b^-based CA^c^	Studies limited to questionnaire development and validation, or solely focused on usability evaluations (primarily emphasizing design factors affecting user-friendliness and user experience) [23], without any acceptance outcomes indicating users’ intention to use a digital tool [22, 24]
The DH intervention emphasized self-management of NCDs or chronic diseases	Studies on physically embodied robots (unless digital robot-like avatars which are available on computers or mobile devices), social media (e.g., Facebook community groups, health forums, websites, etc.), or telephone-based interventions, and telehealth relying solely on online video consultations with human healthcare professionals without the involvement of any e-health^d^ apps
Empirical peer-reviewed scholarly articles published within the last 15 years (approx.) between January 1, 2010 and May 31, 2024, particularly corresponding to the substantial rise in CAs after 2010 [29]	Studies focusing on communicable diseases (e.g., COVID-19), substance use disorders, dentistry, surgery, maternity/pregnancy, accidents, and loneliness
Studies with full-text availability in English	Review articles, protocols, position papers, commentaries, editorials, and conference abstracts

^a^DH: digital health

^b^AI: artificial intelligence

^c^CA: conversational agent

^d^e-health: electronic healthcare

### Screening and Selection

**Fig. 1:**
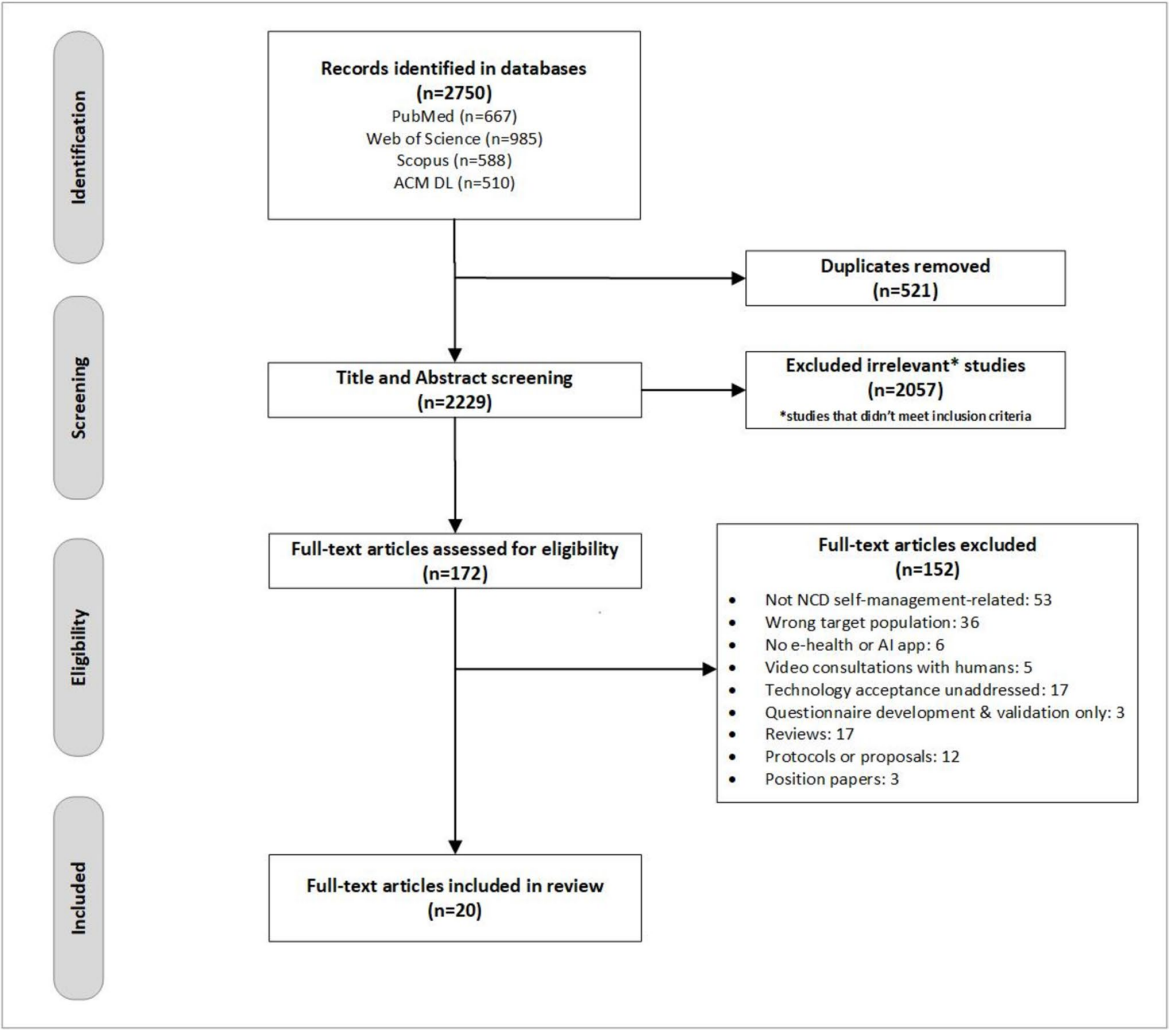
PRISMA (Preferred Reporting Items for Systematic Reviews and Meta-Analyses) flowchart of the search and screening process

Two authors independently searched each database. We used Endnote (Ver. 21) to export articles, and the online Covidence software (SaaS Enterprise) [30, 31] to aid the screening process which was conducted in stages. First, the titles and abstracts of the articles retrieved were screened to identify and remove irrelevant studies based on the predetermined inclusion and exclusion criteria. Abstract screening yielded 172 eligible studies, which were then subjected to full-text screening, when each article was carefully assessed according to predetermined selection criteria and clear reasons for exclusion were recorded. Any discrepancies were resolved through discussion between the authors and consultation with a third reviewer. The search and selection processes are illustrated in Fig. 1.

### Data Extraction and Reporting

The final selection of studies for synthesis was determined by consensus among all the authors after careful assessment of their diverse methodologies for technology acceptance evaluation. Quantitative data, such as statistical measures and acceptance scores, were extracted from included studies alongside qualitative insights derived from thematic analysis, reflecting older adults' intention to use the proposed DH tool for NCD management. The Covidence software [30, 31] facilitated the systematic extraction of quantitative and qualitative data from these studies, which were subsequently summarized in a tabular format. A narrative synthesis approach was used to integrate and analyse the findings, with quantitative data providing measurable insights into perceived acceptability and qualitative data offering a contextual understanding of user attitudes toward DH adoption. The complementary integration of these data types facilitated the identification of main themes related to the acceptance of DH and CA technologies for NCD management among older adults.

## Results

### Characteristics of Included Studies

The 20 included studies in this review were published between 2016 and 2024, with 80% (*n* = 16) in the past 5 years. Information related to characteristics of included studies, such as intervention, participants' demographics, type of technology, sample size, study design and location, measures of technology acceptance, key outcomes, and limitations, are presented in Table 2.

**Table 2:** Included Study Characteristics

Study and Year	Intervention and Study Duration	Technology and Delivery Platform	Target Population, Sample Size, and Participants’ Age	Study design, Framework, and Location	Acceptability Measures and Outcomes	Limitations
Mercer et al., 2016 [49]	• To support self-management of chronic diseases using physical activity trackers • **Duration:** 3–4 months (approx.)	• **Wearables:** A simple pedometer and 4 commercial wearable activity trackers (Fitbit Zip, Misfit Shine, Jawbone Up 24, and Withings Pulse) • **Platform:** Wearable devices connected to smartphones	• Older adults aged (> 50 years) with chronic diseases • N = 32 • **Participants’ Age:** 52–85 years (mean = 64 years)	• **Study type:** Cross-sectional, mixed-methods study • **Instruments:** focus groups and survey questionnaires • **Framework:** TAM^a^ • **Location:** Canada	• **Perceived Usefulness:** Wearable trackers were considered useful and acceptable for increasing physical activity and self-monitoring health • **Perceived Ease of Use:** Concerns about usability; devices were not user-friendly for older adults • **Mean Acceptance Scores:** - Simple Pedometer: 56/95 - Wearable activity trackers: ○ Fitbit: 68 ○ Misfit/Jawbone: 65 ○ Withings: 63 • **Purchase Intentions:** 73% wanted to buy a wearable activity tracker ○ Preference for Fitbit: 50% ○ Misfit/Jawbone/Withings: 42% • **Motivation for Use:** Physician recommendations motivated participants to test the devices	• Small sample size of 32 and cross-sectional study design may limit the generalizability of the findings • Purposive sampling may lead to sampling bias • The study focused on a specific geographic area (southwestern Ontario), which may not represent broader populations • Short testing duration may limit the assessment of long-term adherence and effectiveness • Reliance on self-reported data for physical activity may affect accuracy and bias
Cajita et al., 2017 [43]	• To support self-management of HF^b^ • **Duration:** 6–7 months	• mHealth^c^ technology (e.g., mHealth apps, physical activity tracker wristband, heart rate tracker wristband + heart rate monitoring app, electronic blood pressure cuff) • **Platform:** mobile devices (e.g., wearables, smartphones)	• Older adults (≥ 60 years) with a history of heart failure • N = 129 • **Participants’ Age:** 71.3 ± 4.6 years	• **Study type:** Cross-sectional, correlational, mixed-methods study • **Instruments**: Online and face-to-face survey questionnaires • **Framework**: TAM • **Location:** USA	Statistical significance of the factors affecting users' intention to use mHealth: • **Positive significant correlations:** - Social influence (β = 0.17, P = 0.010) - Perceived ease of use (β = 0.16, *P* < 0.001) - Perceived usefulness (β = 0.33, *P* < 0.001) • **No significant correlations:** - Perceived financial cost (β = − 0.04, P = 0.345) eHealth literacy (β = − 0.01, *P* = 0.799)	• Participants unable to read/understand English or had cognitive impairments were not included, which may limit generalizability • The sample was primarily composed of individuals with at least some college education, which may not represent the broader population of older adults with heart failure • Convenience (non-probabilistic) sampling technique may introduce sampling bias • Reliance on self-reported data may introduce bias
Cajita et al., 2018 [44]	• To support self-management of HF • **Duration:** Not identified	• mHealth technology (e.g., mHealth apps, monitoring systems, wearables) • **Platform:** mobile devices	• Older adults with a history of heart failure • N = 10 • **Participants Age:** ≥ 65 years (not specified)	• **Study type:** Qualitative study • **Instruments**: • Face-to-face in-depth interviews, short surveys, mHealth demonstration, and think-aloud technique for usability testing • **Framework**: TAM • **Location:** USA	• **Facilitators:** familiarity with mobile technology, willingness to learn, ease of use, presence of useful features, adequate training, free equipment, and doctor’s recommendation • **Barriers:** limited literacy of using mHealth, decreased sensory perception, lack of need for technology, poorly designed interface, cost of technology, and limited/fixed income Overall, older adults showed a willingness to use mobile health technology, albeit with reservations	• Small sample size of only 10 participants may limit generalizability of findings • Purposive sampling may lead to sampling bias • The study was confined to one geographical setting (an urban area in the United States), which may limit generalizability • Potential bias from the researchers' beliefs and assumptions • The qualitative nature of the study may not provide statistical significance or quantifiable data
Still et al., 2018 [37]	• To support self-management of hypertension • **Duration:** 2 months (approx.)	• Mobile apps and SMS text reminders • **Platform:** mobile devices	• African American older adults (≥ 60 years) with hypertension • N = 21 • **Participants’ Age:** 62—91 years (mean = 72 years)	• **Study type:** Qualitative study • **Instruments**: focus groups • **Framework**: MoHTAM^d^ • **Location:** USA	• **Perceived Ease of Use:** Simplicity and user-friendliness of mobile health technologies significantly influenced their acceptance • **Perceived Usefulness:** Participants’ belief in the effective assistance of mHealth technology in managing their health was a strong motivator • **Positive Impact on Acceptance:** High perceptions of both ease of use and usefulness with a greater likelihood of acceptance and intention to use technology for health care • **Need for Tailored Interventions:** Development of customized interventions are essential to meet the specific needs and preferences of older adults to facilitate technology acceptance • **Engagement with Technology:** Greater technology engagement (e.g., through social media or communication) led to increased likelihood of accepting digital health interventions • **Barriers to Acceptance:** Concerns included lack of familiarity, privacy issues, and technical difficulties	• Small sample size of 21 participants may limit generalizability of findings • Convenience sampling predominantly with women participants from one geographical location, can lead to sampling bias • The study did not capture male participants’ perspectives adequately, limiting generalizability • Existing demographic data did not allow for further interpretation regarding socioeconomic status • The qualitative inquiry was based on one focus group, which did not allow for data saturation
Jiang et al., 2019 [40]	• To support self-management of CVDs^e^ • **Duration:** Not identified	• Internet-based e-Health or mHealth apps • **Platform:** mobile devices and internet technology	• Adults with chronic CVDs • N = 231 (older adults ≥ 50 years = 178) • **Participants Age:** multiple age groups (18–49, 50–64, 65–74, ≥ 75 years)	• **Study type:** Cross-sectional quantitative study • **Instruments**: Face-to-face interviews with paper-and-pencil questionnaire **Framework**: Multivariate logistic regression model *(no standard theoretical framework used)* • **Location:** China (Beijing)	Statistical significance of the factors affecting users' intention to use mHealth: • **Willingness to Use:** 68.0% of participants were willing to use mHealth; 40.8% of those willing were aged ≥ 65 years • **Age and IT skill:** Older age correlated with lower willingness (*P* < 0.001), due to technical literacy (IT skill), which is the sole significant indicator of willingness to use mHealth (not age) (*P* = 0.02) • **Education and Employment:** Higher education (*P* < 0.001) and being employed (P = 0.001) were associated with greater willingness to use mHealth • **Internet Searching:** Frequent health information seekers were more willing to use mHealth (*P* < 0.001); older adults searched for health-related information on the internet less frequently • **Hospital visits and Disease management motivation:** Over 66.1% of patients aged > 65 years visited hospitals monthly (P = 0.004), indicating a higher motivation for disease management among older adults • **Self-Management Capabilities:** The combined score for self-management at home had no significant correlation with age (P = 0.28) • **Barriers:** 32.0% (mostly from older age groups) were not interested; main reasons included inability to use devices (64.4%) and complexity (16.4%)	• The questionnaire used was newly designed and may not have the same weight as validated instruments from prior studies, indicating a need for further validation • No standard theoretical framework or model used for assessment of technology acceptance or willingness to use mHealth • The study focused on participants' willingness to use mHealth from a broad perspective, rather than evaluating any specific mHealth application
Jeffrey et al., 2019 [32]	• To support the self-management of T2DM^f^ • **Duration:** 5–6 months	• e-health or mHealth apps • **Platform:** mobiles or smartphones	• Adults with a self-reported diagnosis of Type 2 T2DM for > 6 months • N = 30 (16 app users, 14 non-app users) o 18 older adult participants (≥ 50 years) • **Participants Age:** multiple age groups (30–39 years, 40–49 years, 50–59 years, 60–69 years, 70–79 years)	• **Study type:** Qualitative study • **Instruments**: Semi-structured phone interviews • **Framework**: TAM, HI-TAM^g^, and MARS^h^ • **Location:** Australia	• **Improved Self-Management:** Older adult participants reported enhanced self-management behaviors when using mobile phone applications for diabetes management • **Perceived Ease of Use:** Many participants in this age group expressed that the apps were user-friendly, contributing to their willingness to engage with the technology • **Perceived Usefulness:** Older adults found the apps to be beneficial in managing their diabetes, particularly in tracking diet and exercise, • **Motivation and Engagement:** The use of apps increased motivation among older participants to adhere to their diabetes management plans • **Acceptance Barriers:** - Some older participants faced technological issues, such as difficulties with app navigation and connectivity problems, which influenced their overall experience - Lack of recommendations from healthcare professionals was noted as a barrier to app usage among older adults, affecting their confidence in using the technology	• Small sample size may not represent the broader population of individuals with T2DM • Limited generalizability due to the focus on rural populations in Australia • Inclusion of multiple age groups in the study may have diluted the focus on older adult participants, potentially overlooking specific needs and preferences unique to this demographic • Potential bias in self-reported data from participants • Variability in app usage and experiences among participants may influence technology acceptance factors
Woo & Dowding, 2020 [45]	• To support remote health monitoring in HF patients • **Duration:** Not identified	• Telehealth remote monitoring systems • **Platform:** telecommunication technologies	• Patients diagnosed with HF who are at risk for hospitalization • N = 20 (interview participants) • **Participants Age:** mean age = 72.6 years (S.D. 13.4 years), median age = 72.5 years	• **Study type:** Qualitative study • **Instruments**: Non-directive, semi-structured telephone interviews • **Framework**: UTAUT^i^ • **Location:** USA	• **Performance Expectancy:** Participants perceived telehealth technology as beneficial for managing their health, which positively influenced their decision to initiate telehealth services • **Facilitating Conditions:** Access to clinical and technical support increased confidence in using telehealth • **Social Influence:** Support from family and healthcare providers positively influenced adoption decisions • **Effort Expectancy:** Ease of use did not significantly impact the decision to initiate telehealth • **Experience with Telehealth:** Prior experience increased likelihood of adoption • **Knowledge of HF and Telehealth:** Greater knowledge facilitated willingness to engage with telehealth	• Small sample size and specific demographic characteristics of participants, may limit generalizability • Reliance on self-reported data may introduce bias • The study was conducted in a single geographic location, which may not reflect broader trends
Sin et al., 2020 [33]	• To support self-management of T2DM and hypertension • **Duration:** 9 days	• Tele-monitoring (TM) systems • **Platform:** Not specified	• Individuals with self-reported depression T2DM and hypertension • N = 899 • **Participants Age:** 58 ± 8 years	• **Study type:** Quantitative; cross-sectional, observational study • **Instruments**: Face-to-face interviews with interviewer-administered questionnaires • **Framework**: HI-TAM • **Location:** Singapore	Results of HI-TAM-related factors influencing willingness to use TM^j^: • **Behavioural Beliefs (Health Status):** *- Years since diagnosis of diabetes mellitus:* ○ 5 years or less: Out of 210 patients, 123 (58.6%) were willing to use TM (Odds Ratio: 1.672, 95% CI: 1.139–2.457, *p* < 0.01) ○ More than 5 years (reference group): Out of 214 patients, 98 (45.8%) were willing to use TM *- Years since diagnosis of hypertension:* ○ 5 years or less: Out of 298 patients, 167 (56.0%) were willing to use TM (Odds Ratio: 1.298, 95% CI: 1.020–1.745, p = 0.10) ○ More than 5 years (reference group): Out of 434 patients, 131 (30.2%) were willing to use TM • **Normative Beliefs (Subjective Norms)** *- Willingness to use TM after seeing benefits from reports:* ○ More willing: Out of 532 patients, 309 (58.1%) were more willing to use TM (Odds Ratio: 1.734, 95% CI: 1.326–2.268, *p* < 0.01) ○ Not more willing (reference group): Out of 367 patients, 163 (44.4%) were not more willing to use TM *- Concerns about tele-monitoring violating patients’ privacy:* ○ Agree (reference group): Out of 204 patients, 90 (44.1%) were willing to use TM • Disagree: Out of 695 patients, 382 (55.0%) were willing to use TM (Odds Ratio: 0.392, 95% CI: 0.287–0.537, *p* < 0.01) • **Technology Use:** - Ownership of a hand-phone or smartphone was associated with increased willingness to use TM - Self-reported computer skills were also positively associated with willingness to use TM	• Self-reported willingness to adopt TM may not correspond to actual utility by the patients • Short study duration for TM acceptance assessment • The study excluded patients with prior exposure to tele-monitoring and those who could not respond to the survey due to language, cognitive, hearing, or visual barriers • The study was conducted in a specific geographical area (northeastern Singapore), which may limit the generalizability of the findings to other regions or populations
Infarinato et al., 2020 [50]	• To support constant health monitoring and management of chronic diseases or age-related impairments • **Duration:** 4 weeks	• eWALL – home-based health monitoring system prototype • **Platform:** centralized, cloud-based server architecture	• Older adults with chronic conditions (COPD^k^ or MCI^l^) or with an age-related impairment • N = 48 • **Participants Age:** > 65 years (not specified)	• **Study type:** Mixed-methods, longitudinal, observational study • **Instruments**: Online qualitative interviews, quantitative surveys, and data logs • **Framework**: TAM • **Location:** Europe (Austria, Denmark, Italy, and the Netherlands)	Backward Linear Regression Analyses on the dependent variables of TAM factors: • **Ease of Use:** - Model R^2^ = 0.81 - Significant predictors: ○ Aesthetics: β = 0.38, t = 3.48, *p *< 0.01 ○ Controllability: β = 0.59, t = 0.37, *p *< 0.001 • **Perceived Usefulness:** - Model R^2^ = 0.50 - Significant predictor: ○ Trust in technology: β = 0.52, t = 2.56, *p* < 0.05 - Included predictors (not significant): ○ Enjoyment: t = 0.75, p = 0.46 ○ Ease of use: t = 0.37, p = 0.71 ○ Controllability: t = 0.18, p = 0.86 • **Intention to Use:** - Model R^2^ = 0.58 - Significant predictors: ○ Ease of use: β = 0.28, t = 2.08, *p* < 0.05 ○ Perceived usefulness: β = 0.57, t = 4.28, *p* < 0.001 • **Influence of Aesthetics on Enjoyment:** ­ Significant association: β = 0.85, t = 9.28, *p *< 0.001	• The sample size was not large enough to control for both medical and cultural factors simultaneously • Short testing duration of 4 weeks for technology acceptance assessment • The study included participants with different medical backgrounds and from different countries, which may have influenced the results due to cultural and medical heterogeneity • Frequent technological problems while interacting with eWALL, may have hindered participants' use and influenced their opinion and acceptance of the technology
Ahmad et al., 2020 [34]	• To support self-management of T2DM • **Duration:** Not identified	• DH^m^ wearables • **Platform:** Medical wearable devices connected via smartphones	• elderly diabetic patients (≥ 60 years) • N = 223 • **Participants Age:** 60–69	• **Study type:** Quantitative study • **Instruments**: Face-to-face surveys using paper-based survey questionnaire • **Framework**: TAM2^n^ • **Location:** Bangladesh	• Statistical positive significance of 6 constructs on elderly diabetic patients’ continuance intention (CI) to use digital health wearables: – **Perceived usefulness** (β = 0.183, *p* < 0.05) – **Perceived ease of use** (β = 0.165, *p* < 0.05) – **Perceived irreplaceability** (β = 0.138, *p* < 0.05) – **Perceived credibility** (β = 0.165, p < 0.05) – **Compatibility** (β = 0.285, *p* < 0.05) – **Social influence** (β = 0.226, *p* < 0.05)	• **Sample Selection:** Only age was considered when selecting elderly diabetic patients, potentially overlooking other relevant variables such as gender, academic qualification, and marital status • **Geographical Limitation:** Data collected only from Bangladesh, which may limit the generalizability of the findings to other countries with different socioeconomic structures • **Constructs Considered:** The study’s focus limited to only three unique constructs (perceived irreplaceability, perceived credibility, and compatibility), overlooking other potentially impactful constructs like health literacy and health belief
Wong et al., 2021 [39]	• To support self-management of MetS^o^ through a lifestyle intervention program • **Duration:** 3 months	• mHealth app (vs. a booklet) • **Platform:** Mobiles or smartphones	• Older adults MetS, specifically ethnic Chinese smartphone users • N = 77 (app group = 38; booklet group = 39) • **Participants’ Age:** ≥ 50 years (not specified)	• **Study type:** Prospective pilot RCT^p^, mixed-methods study • **Instruments**: Structured questionnaires and health assessments • **Framework**: No standard theoretical framework or model used • **Location:** China (Hong Kong)	• **Intervention group** (App users) showed: - Significant reduction in body weight (β = − 1.069, p = 0.012) - Significant reduction in body mass index (β = − 0.371, p = 0.026) - Greater amount of exercise (β = 8.454, p = 0.032) - Improved exercise self-efficacy (β = 10.62, p = 0.001) • No significant differences between groups for other outcomes • Participants appreciated the intervention, with high acceptability rates (81.58% in the app group and 84.62% in the booklet group)	• Small sample size (77 participants) • Short study duration of follow-up (3 months) for an intervention • Potential self-selection bias in participant recruitment • Limited generalizability due to the specific demographic (ethnic Chinese older adults) • No standard theoretical framework or model used for assessment of technology acceptance or willingness to use the mHealth app
Elnaggar et al., 2021 [41]	• To support self-management of physical activity after cardiac rehabilitation • **Duration:** 2 months (approx.)	• Fitbit Charge 2 (wearable activity tracker), Movn^q^ (mHealth app), and push messages • **Platform:** an mHealth app and a wearable device that syncs with smartphones	• Older adults post cardiac rehabilitation • N = 26 (7 (interviewees) • **Participants Age:** ≥ 60 years (mean age = 66.7 years, SD 8.6)	• **Study type:** RCT, mixed-methods study • **Instruments**: Individual semi-structured interviews, and satisfaction questionnaires • **Framework**: No standard theoretical framework or model used • **Location:** USA	• High satisfaction scores for Fitbit (4.86/5) and Movn mobile app (4.5/5) • Relatively lower satisfaction score for push messages (3.14/5) • Four main themes identified: ­ Technology use increased motivation to be physically active ­ Technology served as a reminder to be physically active ­ Recommendations for technology to improve user experience ­ Desire for personal feedback	• Small sample size (only 7 interview participants) • Limited representation of diverse racial groups and women (mostly White individuals and men) • Recruitment from a single institution may not represent a broader population • Long-term behavioral changes post-intervention are unknown • No standard theoretical framework or model used for assessment of technology acceptance or willingness to use Fitbit and Movn app
Jiwani et al., 2021 [35]	• To support self-management of T2DM through behavioral lifestyle intervention • **Duration:** 6 months	• Fitbit wearable tracker, and companion mHealth app • **Platform:** wearable device connected to smartphones	• older adults (≥ 65 years) with T2DM • N = 18 • **Participants Age:** 72 ± 5.4 years	• **Study type:** Qualitative pilot study • **Instruments**: Focus group interviews conducted online (via WebEx) and in-person sessions • **Framework**: No specific theoretical or model framework used • **Location:** USA	Analysis of acceptability and experience of behavioral lifestyle intervention using Fitbit based on 6 themes: • **High Acceptability:** Participants found Fitbit technology useful and acceptable for diabetes management • **Enhanced Self-Monitoring:** Fitbit improved tracking of diet and physical activity • **Increased Motivation:** Fitbit motivated participants to engage in healthier behaviors • **Intention to Continue Use:** Many participants expressed intention to continue using Fitbit after the study • **Adaptation to Challenges:** Participants adapted their use of Fitbit to overcome COVID-19-related challenges • **Positive Impact on Quality of Life:** The intervention improved participants' overall quality of life	• Small sample size may limit generalizability of findings • Lack of a control group to compare the intervention with and without the Fitbit device • Different experiences due to the adaptation of the intervention delivery method because of COVID-19 • The average age of participants being 72 years, which may limit the transferability of findings to older adults above this age • Potential bias in qualitative interviewing, where participants may have shared more positive experiences than negative ones • No standard theoretical framework or model used for assessment of technology acceptance or willingness to use Fitbit technology
Greer & Abel, 2022 [38]	• To support self-management of hypertension • **Duration:** Not identified	• Commercial mHealth apps • **Platform:** Mobiles or smartphones	• Older Black adults (≥ 50 years) living in rural areas • N = 30 • **Participants Age:** 50–87 years (mean 66.3 ± 9.6 years)	• **Study type:** Convergent parallel mixed-method design • **Instruments**: face-to-face surveys, and focus groups • **Framework**: Adapted TAM • **Location:** USA	• Statistical significance of TAM constructs: **- Perceived ease of use** was significantly correlated with behavioral intention (r = 0.654, *p* < 0.000) **- Perceived usefulness** was also significantly correlated with perceived ease of use (r = 0.585, *p* < 0.001) **- Behavioral intention** was negatively associated with age (r = − 0.047, p = 0.009) • Greater participant adherence was noted with the Hill-Bone Compliance scale (63.3%) compared to the Krousel-Wood scale (23.3%)	• Small sample size may limit the generalizability of findings • Purposive sampling can cause sampling bias • Overrepresentation of older women leading to self-selection bias, as men were underrepresented in the study • Geographical location limits generalizability to older adults in other settings • No comparison group to determine if findings were specific to age or rural location • Limited digital literacy among participants affecting the usability and acceptability of mHealth apps
Nathania et al., 2022 [42]	• To support self-management of AF^r^ through education, monitoring, and self-management features • **Duration:** 6 weeks	• SETAF^s^ mHealth app, integrated within the Philips Motiva system • **Platform:** Tablets with android software, which allows access to the SETAF app	• People diagnosed with AF for 1–25 years • N = 37 (survey = 33; interview = 19) • **Participants Age:** 41–78 (mean age = 65.1 years; with mostly older adults ≥ 50 years)	• **Study type:** Mixed-methods design, with qualitative description to explore patients’ experience with SETAF app using a 21 item SRQR^t^ • **Instruments**: Semi-structured in-depth interviews and a short 4-item survey • **Framework**: TAM • **Location:** Singapore	• **Perceived Usefulness:** *- Usefulness of SETAF in managing participants' conditions:* 21% strongly agreed, 73% agreed, and 6% were neutral - Participants found SETAF valuable for improving their understanding and self-management of AF conditions • **Ease of Use:** The app was reported to be user-friendly, with participants appreciating the guided tutorials that facilitated navigation • **Behavioral Intention to Use:** - 94% of participants reported using SETAF almost every day (6–7 days a week) *- Continuation of use after the study:* 21% strongly agreed, 49% agreed, 24% were neutral, and 6% disagreed *- Willingness to Pay:* Participants expressed a willingness to pay an average of $112.08 SGD for continued access to SETAF, indicating perceived value • **Feedback for Improvement:** Participants provided insights on features they found useful and suggestions for enhancements, which can guide future mHealth interventions	• Limited sample size may limit generalizability of findings • The study did not capture perspectives of non-English speaking AF patients • Improvements in AF knowledge and self-management were self-reported without objective measurements • Short study duration of 6 weeks for assessing acceptance of mHealth app • The study's focus was limited to exploring acceptability and usefulness, rather than assessing the effectiveness of the mHealth tool • While the majority of participants were older adults, the inclusion of a broader age range may affect the findings specifically related to older adults
Haug et al., 2022 [48]	• To support self-management of cognitive health through early detection of Alzheimer’s disease symptoms • **Duration:** 2–3 months	• mHealth app prototype • **Platform:** Mobiles or smartphones	• Seniors with Alzheimer’s disease symptoms • N = 10 • **Participants Age:** 61–93 years	• **Study type:** Qualitative study • **Instruments**: Face-to-face interviews, and participatory workshops • **Framework**: TAM and HBM^u^ • **Location:** Germany	• Privacy concerns were identified as a significant negative influence on technology acceptance • Perceived usefulness was found to play a major role in the willingness to use the application, particularly related to its core functionality • Perceived ease of use was surprisingly not found to be as influential as expected	• Small Sample Size (10 participants) may limit the generalizability of the findings • Qualitative nature may limit the ability to make strong claims about effect sizes and introduce biases • Limited demographic scope (only seniors in Germany), may not reflect other cultural contexts • Lack of longitudinal data on application use and effectiveness • No investigation of how privacy concerns vary among individuals • Lacked comprehensive analysis of necessary facilitating conditions for adoption • Study limited to only assessment of intention to use, not actual usage
Arnaert et al., 2023 [46]	• To support self-management of COPD • **Duration:** 5 months	• Apple Watch Series 6 • **Platform:** Smartwatch connected to smartphones	• Community-dwelling older adults with COPD • N = 10 • **Participants Age:** 64–82 years	• **Study type:** Qualitative descriptive study • **Instruments**: Semi-structured online interviews (via Zoom) • **Framework**: UTAUT2^v^ • **Location:** Canada	Qualitative results of UTAUT2 factors: • **Performance Expectancy:** - Some participants reported health benefits and found the Apple Watch reliable for health monitoring - Others preferred manual oximeters during respiratory distress, indicating mixed perceptions of the Watch's effectiveness • **Effort Expectancy:** - Participants experienced a range of emotions, from curiosity to frustration, due to difficulties in navigating the smartwatch - The device's complexity affected some participants' willingness to engage with its features • **Social Influence:** - Family support significantly encouraged participants to use the Watch, with some relying on family members for assistance • **Facilitating Conditions:** - Participants who owned the Watch were more inclined to explore its features, while non-owners faced limitations in engagement • **Hedonic Motivation:** - The Apple Watch was primarily viewed as a medical tool, but some participants enjoyed its novelty and interaction, contributing to acceptance • **Price Value** - Concerns about the smartwatch's cost were significant for participants, many of whom were on fixed incomes, affecting their perception of its value	• Small sample size (10 participants) may limit generalizability of findings • The majority of participants were female, leading to potential bias • Participants may have had a baseline level of curiosity and comfort with technology, which could bias the results • The Apple Watches were lent to participants, which may have limited their exploration of the device's full capabilities due to fear of damage • Participants only had 5 months to use the Watch, which may have influenced their acceptance • Subjectivity in qualitative research may affect the trustworthiness of the findings
Koo et al., 2023 [51]	• To support health management in older adults with chronic diseases **Duration:** 8 days	• Personalized mHealth service app and wearables • **Platform:** wearable devices connected via smartphones	• Older adults (≥ 60 years) vulnerable to environmental risk factors • N = 477 (278 with at least 1 chronic disease) • **Participants Age:** 60–75 years	• **Study type:** Cross-sectional, quantitative study • **Instruments**: Web-based surveys • **Framework**: UTAUT2 • **Location:** South Korea	• Statistical significance of UTAUT2 factors: **- Performance Expectancy:** Significant predictor of behavioral intention (β = 0.453, *p *< 0.003) - **Social Influence:** Significant predictor of behavioral intention (β = 0.693, *p *< 0.001) **- Facilitating Conditions:** Significant indirect effect on behavioral intention (β = 0.325, *p* = 0.006, 95% CI: 0.115–0.759) • **Device Trust**: Significant indirect effect on behavioral intention in patients with chronic disease (β = 0.122, p = 0.039, 95% CI: 0.007–0.346)	• Cross-sectional design can limit generalizability of findings • Short study duration for assessing technology acceptance • Lack of random sampling (convenience sampling) can lead to sampling bias • Broad definition of "chronic disease" may affect the specificity of the findings • A specific chronic disease was not targeted, which may limit the applicability of the results to particular health conditions • Ambiguities in the comparison of correlation coefficients might affect the validity of the findings
Yu et al., 2023 [36]	• To support self-management of T2DM • **Duration:** 1 year (approx..)	• IMTOP^w^ mHealth app • **Platform:** Mobiles or smartphones, tablets	• Chinese and Hispanic immigrants (mostly older adults) with type 2 diabetes • N = 118 (24 interview participants) • **Participants Age:** mean age = 61.05 years (SD 11.87 years); interviewees' mean age = 63.5 years	• **Study type:** Qualitative study • **Instruments**: face-to-face surveys and semi-structured interviews • **Framework**: UTAUT • **Location:** USA	Qualitative results of UTAUT factors: • **Performance Expectancy:** Participants viewed the app as useful for diabetes management and tracking health behaviors • **Effort Expectancy:** Some found the app easy to use, while others worried about its complexity • **Social Influence:** Family support significantly encouraged participants' intention to use the app • **Facilitating Conditions:** Adequate resources and support were considered essential for adoption • **Intrinsic Motivation:** Strong personal motivation to manage health positively influenced app acceptance • **Intention to Use:** Many participants expressed a strong intention to use the app for self-management • **Barrier Conditions:** Poor eyesight and lack of technical support were common obstacles	• Limited sample size (24 interviewees) may affect the generalizability of technology adoption findings • Purposive sampling may lead to sampling bias • Findings may not apply to immigrants outside major US metropolitan areas • Transcripts were translated to English, potentially losing contextual nuances • Data from 2017–2018 may not reflect current technology adoption trends • Directed content analysis may introduce subjectivity • Reliance on qualitative methods may limit quantification of results • Factors not included in the UTAUT model may have been overlooked in the interviews
Bults et al., 2024 [47]	• To support self-management of dementia • **Duration:** 14 months	• commercial CA^x^—humanlike digital avatar (Anne4Care), which supports video calls • **Platform:** Mobiles or smartphones, tablets	• Older adult immigrants with dementia • N = 13 • **Participants Age:** 52–83 years	• **Study type:** Qualitative descriptive study with naturalistic inquiry (Citizen Science approach) • **Instruments**: Face-to-face in-depth semi-structured interviews • **Framework**: No specific theoretical or model framework used • **Location:** Netherlands	• **High Acceptance:** Participants showed a relatively high acceptance of Anne4Care despite having no prior experience with digital assistive technologies • **Primary Use:** Anne4Care was mainly utilized as a memory assistive tool for managing appointments and medication reminders • **Companionship and Communication:** Participants noted that Anne4Care provided companionship and facilitated communication in multiple languages, which was particularly beneficial for older adult immigrants • **Reduction in Tasks:** Most health care professionals and families reported that Anne4Care helped reduce their tasks and stress levels after the initial setup • **Anxiety and Challenges:** Some participants expressed anxiety regarding the use of Anne4Care and faced challenges with certain functionalities, such as video calling and interacting with the avatar • **Internet Dependency:** The platform required an internet connection at home, limiting its use outside the home • **Improvement Suggestions:** Participants suggested enhancements, such as mobile compatibility for medication reminders when outside, and additional entertainment options like games and videos	• Small sample size (only 13 participants) may limit depth of analysis, particularly in comparing depression severity classes • Combination of moderate and moderately severe depression groups may restrict findings; a four-class comparison could yield more valuable data • Focus on VH mediation may have overshadowed other factors influencing user engagement and task performance • Long-term effects of VH-mediated tasks on user engagement and mental health outcomes were unexplored, limiting insights into intervention sustainability

^a^TAM: Technology Acceptance Model

^b^HF: Heart Failure

^c^mHealth: mobile health

^d^MoHTAM: Mobile Health Technology Acceptance Model

^e^CVDs: Cardiovascular diseases

^f^T2DM: Type 2 Diabetes Mellitus

^g^HI-TAM: Health Information Technology Acceptance Model

^h^MARS: Mobile App Rating Scale

^i^UTAUT: Unified Theory of Acceptance and Use of technology

^j^TM: Tele-monitoring systems

^k^COPD: Chronic Obstructive Pulmonary Disease

^l^MCI: mild cognitive impairment

^m^DH: digital health

^n^TAM2: Extended Technology Acceptance Model

^o^MetS: metabolic syndrome

^p^RCT: randomized controlled trial

^q^Movn: Moving Analytics

^r^AF: Atrial Fibrillation

^s^SETAF: Self-management and Educational technology support Tool for AF patients

^t^SRQR: Standards for Reporting Qualitative Research

^u^HBM: Health Belief Model

^v^UTAUT2: Extended Unified Theory of Acceptance and Use of technology

^w^IMTOP: Intergenerational Mobile Technology Opportunities Program

^x^CA: conversational agent

### Health Interventions

A large proportion of the included studies mainly focused on digital lifestyle interventions aimed at supporting health monitoring and self-management of **T2DM** (5/20; 25%) [32–36], **hypertension** (3/20; 15%) [33, 37, 38], and **MetS** (1/20; 5%) [39]. Some studies (7/20; 35%) emphasized telemonitoring and self-management of **CVDs** [40, 41] (6/20; 30%), including AF [42], heart failure (HF) [43–45], and chronic obstructive pulmonary disease (COPD) [46]. Two studies (10%) addressed technology acceptance for managing cognitive impairments, such as **dementia** [47] and **Alzheimer’s disease** [48], while the remaining (3/20; 15%) explored technology acceptability for managing chronic diseases more generally instead of focusing on any particular NCD [49–51].

### Types of DH Technologies

- ***Mobile Health (mHealth) apps:*** Most of the studies (13/20; 65%) focused on mHealth apps, such as Moving Analytics (Movn) [41], Intergenerational Mobile Technology Opportunities Program (IMTOP) [36], and Self-management and Educational Technology Support Tool for AF patients (SETAF) [42], which are typically accessed via smartphones and tablets. Many of them (6/13; 46%) utilized commercial mHealth app probes without specifying the app names [32, 37, 38, 40, 44, 51], while one evaluated a prototype’s acceptability for early diagnosis of Alzheimer’s disease [48].
- ***Wearable Trackers:*** Some studies (6/20, 30%) used wearable tracking devices, including Apple Watch® [46], Fitbit® [35, 41, 49], Misfit Shine, Jawbone Up 24, Withings Pulse, and a simple pedometer [49]. A few studies (3/20; 15%) also used unspecified commercial wearables [34, 44].
- ***Tele-monitoring systems****:* Three studies explored telehealth monitoring systems [33, 45], including eWALL, a home-based health monitoring system prototype designed to monitor users' physical and cognitive behaviors [50].
- ***Conversational Agent (CA)****:* One study (1/20; 5%) investigated the acceptance of Anne4Care, a human-like, voice-activated CA avatar accessible via smartphones and tablets, designed to support dementia self-management among older adult immigrants [47]. While the CA is expected to be AI-based, the study did not specify AI integration.

### Study Durations

The study durations ranged from 8 days to 14 months. Short-term durations included 8–9 days [33, 51] and 4–6 weeks [42, 50], while intermediate durations spanned 2–4 months [37, 39, 41, 48, 49]. Longer durations extended from 5–7 months [32, 35, 43, 46], and up to 12–14 months [36, 47]. The remaining studies (5/20; 25%) did not specify durations [34, 38, 40, 44, 45].

### Study Populations

The target population consisted of older adults (≥ 50 years) with chronic diseases, ensuring comprehensive understanding across an inclusive age range. Half of the included studies (10/20; 50%) specifically focused on participants aged 60 years and above. However, a few studies (3/20; 15%) did not restrict age and included a broad range of participants, encompassing younger and middle-aged adults [32, 40, 42].

### Locations

The majority of the studies (19/20; 95%) were conducted in high-income and upper-middle-income countries. Half occurred in North America – 8 (40%) in the USA, and 2 (10%) in Canada. Three studies (15%) were conducted in Europe: Austria, Denmark, Italy, the Netherlands [47, 50], and Germany [48]. One study (5%) was performed in rural Australia [32], while the remaining six (30%) were conducted in Asia, including Singapore [33, 42], South Korea [51], China [39, 40], and Bangladesh (the only low-income country) [34].

### Study Designs

Nearly half of the studies (9/20; 45%) were qualitative, including three qualitative descriptive studies [45–47] and one pilot study [35]. The instruments included focus groups (*n* = 2) [35, 37], interviews (*n* = 4) [32, 45–47], short surveys (*n* = 1) [42], participatory workshops (*n* = 1) [48], and usability testing using the think-aloud technique (*n* = 1) [44], all aimed at elucidating user perceptions and technology acceptability for health management.

A considerable portion (7/20; 35%) utilized mixed-methods approaches, comprising two cross-sectional studies with focus groups [49] and surveys [43], two randomized controlled trials (RCTs) [39, 41] — one using structured health assessment questionnaires [39] and the other combining semi-structured interviews and quantitative survey questionnaires for user satisfaction evaluation [41], one convergent parallel design using qualitative focus groups and quantitative health measures [38], and one longitudinal observational study utilizing both quantitative surveys and qualitative interviews [50].

The remaining four studies (20%) adopted quantitative methods, predominantly cross-sectional [40, 51], including one cross-sectional observational study [33], utilizing surveys [34, 51] and interviews as instruments [33, 40].

### Theoretical Frameworks and Influential Factors

The Technology Acceptance Model (TAM) was the most frequently used theoretical frameworks, applied in eleven (55%) studies, including its extended (TAM2) and health-focused modified versions— Health Information Technology Acceptance Model (HI-TAM), Mobile Health Technology Acceptance Model (MoHTAM) [32–34, 37, 38, 42–44, 48–50]. Two studies combined TAM with other relevant models— Health Belief Model (HBM) and Mobile App Rating Scale (MARS) [32, 48]. The Unified Theory of Acceptance and Use of Technology (UTAUT), including its extended version (UTAUT2), was used in four (20%) studies [36, 45, 46, 51]. The remaining five (25%) studies focused on older users’ attitudes towards technology adoption for chronic disease self-management without applying a specific theoretical framework [39–41, 47]. Table 3 presents an overview of these frameworks, highlighting the influential factors (as ***facilitators*** or ***barriers***) based on their importance and prevalence across studies.

- ***Facilitators:*** The primary positive determinants of technology adoption are perceived usefulness (or performance expectancy) [32, 34, 36–38, 42–46, 48–51] and perceived ease of use (or effort expectancy) [32, 34, 36–38, 42–44, 46, 49, 50]. both strongly associated with the intention to use health applications [24, 52–54], though ease of use was found insignificant in two studies [45, 48]. Social influence, especially recommendations from family and healthcare professionals, was another key driver [32, 34, 36, 43–46, 49, 51]. Digital literacy and telehealth familiarity [33, 37, 40, 44, 45], along with motivation and positive attitudes toward technology can further promote behavioral intention to use DH [32, 36, 37, 44, 46, 55], although e-health literacy was insignificant in one study (*P* = 0.799) [43].
- ***Barriers:*** Privacy concerns [33, 37, 48], along with technical challenges and usability issues, including inadequate technical support, connectivity issues, internet dependency and interface navigation complexities, substantially hinder technology acceptance [32, 36, 44, 47, 49]. Sensory impairments (e.g., reduced eyesight) can also impede adoption in older adults [36, 37]. Cost was reported as a barrier in two studies [44, 46], while one study found it insignificant (*P* = 0.345) [43].

**Table 3:** Theoretical frameworks and key influential factors affecting technology acceptance

Theoretical Frameworks	Frequency, n (%)	Influential Factors *(ranked by importance and frequency)*	
		Facilitators *(Factors positively influencing Behavioral Intention to Use Technology)*	Barriers *(Factors negatively influencing Behavioral Intention to Use Technology)*
TAM^a^	6 (30%) [38, 42–44, 49, 50]	1. Perceived Usefulness [32, 34, 37, 38, 42–44, 48–50] 2. Perceived Ease of Use [32, 34, 37, 38, 42–44, 49, 50] 3. Social Influence [32, 34, 43, 44, 49] 4. Digital or e-health^i^ literacy [33, 37, 40, 44] 5. Motivation to use e-health [32, 37, 44] 6. Perceived credibility or trust [34, 50] 7. Controllability [50] 8. Aesthetics [50] 9. Compatibility [34] 10. Perceived irreplaceability [34] 11. Personalization [37]	1. Privacy concerns [33, 37, 48] 2. Technical challenges (e.g., app navigation difficulty, connectivity problems, inadequate technical support) [32, 37] 3. Usability issues (e.g., complex or poorly designed interfaces) [32, 33] 4. Sensory impairments [37] 5. Cost [44]
TAM2^b^	1 (5%) [34]		
HI-TAM^c^	1 (5%) [33]		
TAM + HI-TAM + MARS^d^	1 (5%) [32]		
TAM + HBM^e^	1 (5%) [48]		
MoHTAM^f^	1 (5%) [37]		
UTAUT^g^	2 (10%) [36, 45]	1. Performance expectancy (e.g., perceived usefulness) [36, 45, 46, 51] 2. Social Influence [36, 45, 46, 51] 3. Facilitating conditions (e.g., clinical and technical support access, ownership of DH^j^ devices) [36, 45, 46, 51] 4. Effort expectancy (e.g., perceived ease of use) [36, 46] 5. Hedonic or intrinsic motivation (e.g., novelty, enjoyment, personal interest) [36, 46] 6. Familiarity with Telehealth [45] 7. Device Trust [51]	1. Technical challenges (app complexity, inadequate technical support) [36, 46] 2. Sensory impairments (e.g., poor eyesight) [36] 3. Price value or cost [46]
UTAUT2^h^	2 (10%) [46, 51]		

^a^TAM: Technology Acceptance Model

^b^TAM2: Extended Technology Acceptance Model

^c^HI-TAM: Health Information Technology Acceptance Model

^d^MARS: Mobile App Rating Scale

^e^HBM: Health Belief Model

^f^MoHTAM: Mobile Health Technology Acceptance Model

^g^UTAUT: Unified Theory of Acceptance and Use of technology

^h^UTAUT2: Extended Unified Theory of Acceptance and Use of technology

^i^e-health: electronic healthcare

^j^DH: digital health

## Discussion

### Principal Findings

The discussion in this review synthesizes the prominent findings aligned with each research question, which highlights the perceived acceptability of DH and AI-based CAs, theoretical frameworks, and key influential factors of technology acceptance for NCD management among older adults, as reflected in the reviewed literature.

### Acceptability of DH Technologies Among Older Adults

Most reviewed studies indicated that older adults generally exhibit positive attitudes towards acceptance of DH technologies, which is evidenced both quantitatively through high acceptance scores (≥ 63 out of 95) [49], and the significance of key factors influencing intention to use technology (*p* < 0.05) [33, 38, 40, 43, 50, 51], as well as qualitatively through participants’ favorable comments [35, 36, 46, 47]. Beyond quantitative measures, understanding the nuances of technology acceptance requires insights from qualitative and mixed-method approaches, as utilized in most of the included studies. These approaches provide a deeper understanding of patients' individual experiences and subjective attitudes toward telehealth systems, which can substantially predict their adoption [56]. Likewise, a few related external studies further contributed valuable perspectives on DH acceptability. For instance, Rochmawati et al. (2022) found through a qualitative study that older adults in primary healthcare settings preferred remote e-health monitoring systems for home health management owing to comfort and ease of use [57], which aligns with this demographic’s mobility limitations and the need for user-friendly DH tools to reduce their frequent hospital visits [58]. Conversely, a mixed-methods study by D'Haeseleer et al. (2020) reported low behavioral intention to use self-management e-health systems among healthy older adults, primarily due to a lack of perceived value in these interactive technologies, despite reasonably high levels of perceived ease of use and confidence [53]. These contrasting findings suggest that users’ health status, comfort and perceived usefulness of DH technologies can significantly influence their acceptance.

One notable finding is the limited acceptance evaluation of AI-assisted CAs among older adults for managing NCDs, with only one included study involving a potentially AI-based CA, though not explicitly stated [47]. This inadequate evaluation exists despite the emergence and growing application of AI-based CAs powered by large language models (LLMs) (e.g., GPT series, Llama) in remote care [2], as well as the availability of established methodological frameworks for evaluating technology acceptance (e.g., TAM, UTAUT). This deficiency may be attributed to a paucity of studies employing these frameworks or methodological limitations, such as inadequate expansion or validation of such frameworks tailored to the specific healthcare context addressed in these studies [24]. However, recent studies indicate that older adults may be more inclined to adopt CAs because of their voice-enabled, human-like interactions, associated with improved user-friendliness and trust [18, 59], which resonate with their preferences and needs [2, 29, 60, 61].

### Variation of Technology Acceptance Across NCDs

The review found no clear evidence of variation in perceived technology acceptance across various NCDs (e.g., diabetes, hypertension, MetS, CVDs, and dementia) when managing them with different DH technologies. Notably, most of the NCDs highlighted in these studies were physical chronic diseases, with only a few addressing cognitive conditions like dementia [47] and Alzheimer’s disease [48], both commonly classified as age-related cognitive impairments. In contrast, our recent scoping review focusing on AI-powered CAs for NCD self-management identified a significant proportion of studies targeting mental health issues (e.g., depression and anxiety) predominantly observed in younger populations [2]. This difference may stem from the prioritization of physical health needs as a fundamental concern in older adults [62], leading to a greater perceived value of digital technologies for managing physical or age-related chronic diseases compared to mental health or psychological needs. Nevertheless, it remains crucial to consider the influential factors of technology adoption, both as facilitators and barriers, when developing DH tools to enhance their use and acceptance among older adults for self-managing targeted NCDs.

### Theoretical Frameworks for Technology Acceptance Evaluation

Technology acceptance is a multifaceted assessment influenced by various determinants affecting users' willingness to adopt new technologies [63], often guided by standard theoretical frameworks. The two widely used frameworks are the TAM established by Davis (1986, 1989) [64, 65], and the UTAUT introduced by Venkatesh et al. (2003) [66]. TAM is favored for its simplicity and ease of application, providing quick insights into individual perceptions of technology while demonstrating high explanatory power through extensive validation [67, 68]. UTAUT offers greater explanatory power incorporating elements from eight prior models, including moderating factors such as gender, age, and experience, to provide a more comprehensive understanding of technology acceptance, particularly in organizational settings [69]. Additionally, multiple studies used their extended (e.g., TAM2, UTAUT2) [34, 46, 51] or modified versions (e.g., HI-TAM, MoHTAM) [32, 33, 37] for more tailored evaluations in the healthcare context. Interestingly, the Senior Technology Acceptance Model (STAM), a modified version of TAM designed for older adults, was not utilized in any of the included studies, despite its recognized focus on the perceived usefulness of assistive technologies like wearables, in supporting older adults' independence, safety and health-related outcomes [70, 71]. Nonetheless, STAM's limited expansion on the acceptance of healthcare technologies specifically, coupled with its inadequate consideration of the impact of novelty and complexity on technology acceptance, may have hindered its widespread application [24].

### Influential Factors of Technology Acceptance

Perceived usefulness and ease of use consistently emerge as primary determinants of technology acceptance, particularly in studies applying the TAM and UTAUT models. While TAM emphasizes these factors, UTAUT extends by incorporating performance and effort expectancy respectively, offering a more holistic understanding of acceptance criteria. Social influence, particularly support from family members, caregivers, and healthcare providers, also plays a significant role in both models, enhancing trust and encouraging adoption [32, 34, 44, 45, 49, 51, 57]. Additionally, while digital or e-health literacy [33, 37, 40, 44], and familiarity with telehealth [45] are frequently identified as favourable determinants, positive attitudes [52, 55] and motivation to use mHealth have also been recognized as key facilitators of DH acceptance among older adults, irrespective of digital literacy [32, 36, 37, 44, 46, 59]. Nevertheless, multiple studies have reported an inverse relationship between age and technology use intention, often attributed to limited digital literacy [40, 72, 73]. Older adults, especially in LMICs, generally have less exposure to technology, reducing their inclination toward adoption [74, 75]. Besides, the complexity, internet access, affordability (or cost-effectiveness) of AI-based DH tools, along with the limited availability of Food and Drug Administration (FDA)-approved AI-assisted medical devices tailored to geriatric health needs [76], can further restrict their acceptability among aged populations in LMICs with scarce technological resources and widespread financial constraints [75, 77]. Moreover, concerns about prolonged and repeated use of digital devices for accessing contents, leading to eye strain or an increased risk of Computer Vision Syndrome, can pose challenges in managing regular use of DH tools for older adults, affecting their long-term acceptance [78, 79]. Consequently, targeted awareness and training programs aimed at improving key facilitators, such as perceived usefulness, e-health literacy, and social influence [44], may improve DH acceptance in older adults for NCD management, potentially contributing to mitigating the global health burden.

Beyond the commonly identified factors, anthropomorphism, which enhances human-like interaction in AI CAs (e.g., digital avatars), can make interactions more intuitive and engaging, thus facilitating adoption [2, 61, 80]. However, only one study involved a CA without explicitly evaluating these anthropomorphic characteristics [47]. Besides, factors such as education, employment, hospital visits, health management motivation, technology engagement and companionship, were noted in studies lacking structured theoretical frameworks [35, 40, 41, 47], indicating underexplored practical elements. Furthermore, privacy and data security concerns regarding sensitive health data handling [10, 48, 81], highlight a need for model adaptations to include these factors specific to older adults.

### Implications and Future Recommendations

Our review highlights limited attention to DH acceptance for NCD management among seniors, despite their potential and rapid advancements in healthcare [24, 82]. Recent studies indicate the potential effectiveness of AI-assisted telehealth in non-pharmacological interventions for constant remote management of NCDs [2]. Nevertheless, healthcare professionals often regard lifestyle modifications as challenging treatments due to the need for sustained patient effort and initiative [83]. While many healthcare providers feel capable of discussing lifestyle changes, constraints such as their limited time and perceived patient resistance frequently impede the effective implementation of these interventions for chronic disease management [84, 85].

Patient-centered telehealth with AI integration offers a feasible and cost-effective approach to complement traditional in-person treatments [21, 86]. Evidently, AI-based CAs can mitigate usability challenges in conventional telehealth for older adults by providing voice-activated, user-friendly interfaces that support multilingual, human-like interactions, tailored to specific chronic diseases with cultural and contextual sensitivity to meet older users' personalized needs [2, 37, 41]. Furthermore, AI-based CAs can act as health educators not only to older adult patients but also to the family and other caregivers [87, 88], creating a collaborative environment for patient education and engagement for decision-making and management similar to technology-enhanced collaborative learning which has proven to be highly beneficial [89]. Despite these benefits, the acceptance of AI-based CAs for NCD management among older adults is relatively unknown. Therefore, further research is essential to thoroughly evaluate the acceptance and willingness to use these advanced technologies for health management among older adults who are more susceptible to NCDs and require ongoing healthcare support. This help ensure the long-term effectiveness of such technologies and provide valuable insights to inform designers, developers, and policymakers in implementing inclusive health technologies tailored to the aging population’s needs.

AI algorithms can collect and process vast amounts of sensitive patient data to provide insights into health conditions, predict disease progression, identify high-risk patients, and recommend personalized treatment plans, potentially improving adherence to treatment protocols and enhancing overall health outcomes [2, 9, 90]. However, these advancements also raise significant privacy and security concerns [91], which necessitate robust data protection measures during the development of DH systems [81, 92]. Additionally, AI-generated responses from CAs regarding health management should be supervised by respective healthcare professionals to ensure accuracy and patient safety, preventing the dissemination of inappropriate medical advice. Moreover, assessing healthcare professionals’ perspectives on the appropriateness of technology is also crucial [52], as their recommendations can significantly influence the perceptions of patients often relying on medical advice. Hence, future research needs to investigate healthcare professionals' views on the suitability and effectiveness of DH tools to better support NCD self-management.

### Limitations

Despite this research area’s novelty and our thorough literature search strategy, relevant studies might have been excluded (e.g., those published in non-English languages, not indexed in our searched databases, or overlooked due to unlisted search terms or keywords). Our strict inclusion and exclusion criteria focused on the acceptance of DH, including CA technologies for NCD management among older adults to ensure relevance. However, this may have unintentionally narrowed the scope, potentially missing studies offering broader insights. We aimed for a fair selection to minimize selection bias and maintain a balanced representation, though publication bias and other biases beyond our control may still exist. Additionally, new studies published after our search deadline of June 1, 2024, could introduce further variations in the findings.

### Methodological Limitations

The methodological limitations of included studies may also affect this review’s overall findings:

- ***Small Sample Sizes:*** The majority of the studies (15/20; 75%) involved relatively small participant numbers (< 100) [32, 35, 37–39, 41, 42, 44–50], which may limit the generalizability of findings pertaining to technology acceptance for NCD management in the aging population.
- ***Sampling Bias:*** Many studies utilized purposive or convenience sampling methods, which may not accurately represent the broader population and could lead to biased results [36–38, 43, 44, 49, 51]. Besides, a few studies had homogeneous participant groups (e.g., predominantly female or specific age ranges), which may have overlooked the needs and experiences of other demographic groups [32, 37].
- ***Geographic Limitations:*** A significant constraint is the geographic concentration of studies, predominantly in high-income countries [33, 34, 37, 38, 44, 45, 49]. This geographic bias restricts the generalizability of findings, particularly in LMICs, where healthcare infrastructure, digital literacy, and technology access may differ substantially [74, 93]. The underrepresentation of LMICs emphasizes the necessity for future research in more diverse settings to ensure broader applicability.
- ***Reliance on Self-reported Data:*** Most studies depended on self-reported measures for data collection, introducing potential bias and affecting the accuracy of the findings [32, 33, 42, 45, 49].
- ***Short Study Durations:*** A substantial number of studies had relatively short testing durations for assessing technology acceptance, potentially overlooking insights into long-term acceptance and sustained use of these technologies for health management [33, 39, 42, 49–51]
- ***Inclusion of Broad Age Ranges:*** Some studies included a broad age range, including younger participants, which may dilute the focus on older adults and affect the generalizability of findings specifically for this demographic [32, 40, 42].
- ***Technical Issues and Barriers:*** Participants reported various technological challenges, including usability concerns and lack of familiarity, which could hinder the acceptance and effective use of interventions [32, 50].

## Conclusion

This systematic scoping review uniquely examines DH acceptance among older adults for managing NCDs, beyond feasibility and usability assessments. It comprehensively synthesizes diverse studies, providing broad insights into technology acceptance frameworks and influential factors of DH acceptance among older adults. Facilitators such as perceived usefulness, ease of use, and social influence positively impact their acceptance, while barriers like usability issues, technical challenges, and privacy concerns persist that must be addressed to promote long-term DH adoption.

Our findings disclose generally positive attitudes of older adults towards the adoption of DH technologies for NCD self-management, highlighting their potential to enhance remote care for the aging population. Nevertheless, effective integration of these technologies into healthcare practice requires collaboration among respective stakeholders including healthcare providers, technology developers, and policymakers to establish user-centered designs, comprehensive training programs with supportive resources, and robust data protection measures to facilitate the adoption of DH solutions tailored to the unique needs of the aging population.

Furthermore, continued research, particularly focused on AI-based CAs for specific chronic conditions, is crucial to deepen our understanding of their acceptance and impact. Besides, inclusive research incorporating healthcare professionals' perspectives is highly encouraged, as their insights can substantially shape patients’ perceptions of DH tools. Additionally, researchers are recommended to conduct extensive evaluations of technology acceptance frameworks within this context to ensure the successful implementation of AI-assisted DH solutions. In conclusion, this work serves as a foundational resource for stakeholders seeking to integrate DH technologies into NCD management, eventually improving health outcomes and quality of life for older adults.

## Supplementary Information

Below is the link to the electronic supplementary material.

Supplementary file1 (DOCX 15 KB)

## Data Availability

No datasets were generated or analysed during the current study.

## Abbreviations

AF: Atrial Fibrillation
AI: Artificial Intelligence
CA: Conversational agent
COPD: Chronic Obstructive Pulmonary Disease
CVD: Cardiovascular disease
e-health: Electronic healthcare
DH: Digital health
HF: Heart Failure
HI-TAM: Health Information Technology Acceptance Model
IMTOP: Intergenerational Mobile Technology Opportunities Program
LLM: Large language model
LMIC: Low- and middle-income country
MARS: Mobile App Rating Scale
mHealth app: Mobile health application
MoHTAM: Mobile Health Technology Acceptance Model
PRISMA-ScR: Preferred Reporting Items for Systematic reviews and Meta-Analyses extension for Scoping Review
RCT: Randomized Controlled Trial
SETAF: Self-management and Educational Technology Support Tool for AF patients
STAM: Senior Technology Acceptance Model
SUS: System Usability Scale
T2DM: Type 2 Diabetes Mellitus
TAM: Technology Acceptance Model
TAM2: Extended Technology Acceptance Model
UTAUT: Unified Theory of Acceptance and Use of Technology
UTAUT2: Extended Unified Theory of Acceptance and Use of Technology
