# Supplementary material for: Exploring Acceptance of Digital Health Technologies for Managing Non-Communicable Diseases Among Older Adults: A Systematic Scoping Review
Source: J Med Syst. 2025 Mar 11;49(1):35. doi: 10.1007/s10916-025-02166-3 (PMC11897087; doi:10.1007/s10916-025-02166-3)
Supplement: Supplementary file 1 — Supplementary file1 (DOCX 15 KB) [file 10916_2025_2166_MOESM1_ESM.docx]

**Database Search Strategy**

**PubMed:**

(“technology” AND “acceptance”) OR (“technology” AND “adoption”) OR (“acceptance model” OR “technology acceptance”) OR (“TAM” OR “TAM2” OR “STAM” OR “UTAUT” OR “UTAUT2”) AND (“older adult*” OR “older patient*” OR “elderly” OR “geriatric*” OR “aging” OR “ageing” OR “senior*”) AND (“health*” OR “*care” OR “chronic disease*" OR “non-communicable disease*” OR “noncommunicable disease*”) AND (“self-manage*” OR “self-monitor*” OR “self-care” OR “self-treat*” OR “manage*” OR “monitor*” OR “control*” OR “prevent*” OR “treat*” OR “remote care” OR “intervention”) AND (“artificial intelligen*” OR “intelligent” OR “AI” OR “AI-based” OR “AI-powered” OR “AI-assisted” OR “machine learning” OR “deep learning” OR “chatbot*” OR “conversational agent*” OR “virtual agent*” OR “virtual coach*” OR “virtual assistant*” OR “digital health” OR "internet*" OR "web" OR "mobile*" OR "smartphone*" OR "ubiquitous" OR “e-health” OR “e-Health” OR “mHealth” OR “mhealth” OR “m-health” OR “m-Health” OR "mobile health" OR “telehealth” OR “telemedicine”)

**Web of Science** *(All Databases)***:**

((((ALL=((“technology” AND “acceptance”) OR (“technology” AND “adoption”) OR (“acceptance model” OR “technology acceptance”) OR (“TAM” OR “TAM2” OR “STAM” OR “UTAUT” OR “UTAUT2”) )) AND ALL=((“older adults” OR “older patients” OR “elderly” OR “geriatrics” OR “aging” OR “ageing” OR “seniors”))) AND ALL=((“health” OR “care” OR “chronic diseases" OR “non-communicable diseases” OR “noncommunicable diseases”) )) AND ALL=((“self-manage” OR “self-management” OR “self-managing” OR “self-monitor” OR “self-monitoring” OR “self-care” OR “self-treat” OR “self-treatment” OR “manage” OR "management" OR "managing" OR “monitor” OR "monitoring" OR “control” OR “controlling” OR “prevent” OR “preventing” OR “prevention” OR “treat” OR “treatment” OR “treating” OR “remote care” OR “intervention”) )) AND ALL=((“artificial intelligence” OR “intelligent” OR “AI” OR “AI-based” OR “AI-powered” OR “AI-assisted” OR “machine learning” OR “deep learning” OR “bot” OR “conversational agent*” OR “virtual agent” OR “virtual assistant” OR “digital health” OR "internet" OR "web" OR "mobile" OR "smartphone" OR "ubiquitous" OR “e-health” OR “e-Health” OR “mHealth” OR “mhealth” OR “m-health” OR “m-Health” OR "mobile health" OR “telehealth” OR “telemedicine”))

**Scopus:**

( TITLE-ABS-KEY ( "techn* accepta*" OR "techn* adoption" OR {technology acceptance model} OR "TAM" OR "TAM2" OR "STAM" OR "H?TAM" OR "UTAUT" OR "UTAUT2" ) ) AND ( TITLE-ABS-KEY ( "older adult?" OR "older patient?" OR "elderly" OR "geriatric*" OR "ag?ing" OR "aged" OR "senior?" ) AND PUBYEAR > 2009 AND PUBYEAR < 2025 AND PUBYEAR > 2009 AND PUBYEAR < 2025 ) AND ( TITLE-ABS-KEY ( "health*" OR "*care" OR "chronic disease?" OR "non?communicable disease?" AND "self-manag*" OR "self-monitor*" OR "self?care" OR "self?treat*" OR "manag*" OR "monitor*" OR "control*" OR "prevent*" OR "treat*" OR "remote care" OR "intervention" ) AND PUBYEAR > 2009 AND PUBYEAR < 2025 AND PUBYEAR > 2009 AND PUBYEAR < 2025 ) AND ( TITLE-ABS-KEY ( "artificial intelligen*" OR "intelligen*" OR {AI} OR "AI-based" OR "AI-powered" OR "AI-assisted" OR {machine learning} OR {deep learning} OR "*bot" OR "virtual agent?" OR "virtual coach*" OR "virtual assistant?" OR {digital health} OR "internet*" OR "web*" OR "mobile*" OR "smartphone*" OR "ubiquitous" OR "e?health" OR "e?Health" OR "m?health" OR "m?Health" OR {mobile health} OR "telehealth" OR "telemedicine" ) AND PUBYEAR > 2009 AND PUBYEAR < 2025 AND PUBYEAR > 2009 AND PUBYEAR < 2025 ) AND ( LIMIT-TO ( LANGUAGE , "English" ) )

**ACM Digital Library:**

"query": { ((((ALL=(("technology accepta*") OR ("technology adoption") OR ("technology acceptance model") OR ("TAM" OR "TAM2" OR "STAM" OR "H?TAM" OR "UTAUT?" OR "UTAUT2") )) AND ALL=(("older adult?" OR "older patient?" OR "elderly" OR "geriatric?" OR "ag?ing" OR "senior?"))) AND ALL=(("health*" OR "*care" OR "chronic disease?" OR "non-communicable disease?" OR "noncommunicable disease?") )) AND ALL=(("self-manag*" OR "self-monitor*" OR "self-care" OR "self-treat*" OR "manage*" OR "managing" OR "monitor*" OR "control*" OR "prevent*" OR "treat*" OR "remote care" OR "intervention") )) AND ALL=(("artificial intelligen*" OR "intelligent" OR "AI" OR "AI-based" OR "AI-powered" OR "AI-assisted" OR "machine learning" OR "deep learning" OR "*bot?" OR "virtual agent?" OR "virtual assistant?" OR "digital health" OR "e?health" OR "e?Health" OR "m?Health" OR "m?health" OR "mobile health" OR "telehealth" OR "telemedicine")) }

"filter": { E-Publication Date: (01/01/2010 TO 05/31/2024), ACM Content: DL }
